# Establishment of a PEG-mediated protoplast transformation system based on DNA and CRISPR/Cas9 ribonucleoprotein complexes for banana

**DOI:** 10.1186/s12870-020-02609-8

**Published:** 2020-09-15

**Authors:** Shaoping Wu, Haocheng Zhu, Jinxing Liu, Qiaosong Yang, Xiuhong Shao, Fangcheng Bi, Chunhua Hu, Heqiang Huo, Kunling Chen, Ganjun Yi

**Affiliations:** 1 College of Horticulture, Hunan Agricultural University, Changsha, China; 2 Key Laboratory of South Subtropical Fruit Biology and Genetic Resource Utilization (Ministry of Agriculture and Rural Affairs), Guangdong Key Laboratory of Tropical and Subtropical Fruit Tree Research, Institute of Fruit Tree Research, Guangdong Academy of Agricultural Sciences, Guangzhou, China; 3 State Key Laboratory of Plant Cell and Chromosome Engineering, Center for Genome Editing, Institute of Genetics and Developmental Biology, Chinese Academy of Sciences, Beijing, China; 4 University of Chinese Academy of Sciences, Beijing, China; 5 Mid-Florida Research and Education Center, University of Florida, Apopka, FL USA

**Keywords:** PEG-mediated, Protoplast transformation, Deep amplicon sequencing, Genome editing, DNA-free

## Abstract

**Background:**

To date, CRISPR/Cas9 RNP editing tools have not been applied to the genetic modification of banana. Here, the establishment of a PEG-mediated banana protoplast transformation system makes it possible to build an efficient DNA-free method for a site-directed mutagenesis system.

**Results:**

Protoplasts constitute a versatile platform for transient expression in plant science. In this study, we established a PEG-mediated banana protoplast transformation system. This system was further optimized for successfully delivering CRISPR/Cas9 and CRISPR/Cas12a plasmids and CRISPR/Cas9 ribonucleoproteins (RNPs) for targeted delivery of the *PDS* gene into banana protoplasts. Specific bands were observed in PCR-Restriction Enzyme Digestion (PCR-RE) assays, and Sanger sequencing of single clones further confirmed the occurrence of indels at target sites. Deep amplicon sequencing results showed that the editing efficiency of the CRISPR/Cas9 system was higher than that of the other two systems.

**Conclusions:**

The PEG-mediated banana protoplast transformation system can serve as a rapid and effective tool for transient expression assays and sgRNA validation in banana. The application of the CRISPR/Cas9 RNP system enables the generation of banana plants engineered by DNA-free gene editing.

## Background

A protoplast is a cell of a plant, fungus, bacterium, or archaeon from which the cell wall has been removed by plasmolysis, leaving the protoplasm and plasma membrane. As early as 1892, Klercker obtained protoplasts by a mechanical method [1], which resulted in a low yield and suffered from difficult operation and poor applicability. In 1960, Cocking successfully isolated tomato root tip protoplasts for the first time by enzymatic hydrolysis [2]. This method was widely used because of its high yield, high activity, easy operation and wide adaptability. The transient transformation system of plant protoplasts without unique cell wall characteristics is extensively used in genetic research involving gene function identification, subcellular localization and gene editing. The common methods used for plant protoplast transformation include PEG-mediated transformation [3, 4], electroporation-mediated transformation [5–9] and microinjection-based transformation [10]. Among these, the PEG-mediated method is widely used due to its easy operation, low cost, lack of requirements for specific equipment and generation of stable results. To this date, mature and stable genetic transformation systems for protoplast transient expression have been established in *Arabidopsis* [11–14], wheat [15], rice [16], maize [17] and other species.

Currently, there are many reports on PEG-mediated protoplast transformation of DNA, but few reports on PEG-mediated protoplast transformation of ribonucleoprotein (RNP). In 2015, Woo et al. directly transferred RNP into the protoplasts of *Arabidopsis thaliana*, tobacco and rice for the first time. The genome-edited mutant regenerated from protoplasts contained no transgenic ingredients [18]. In 2016, Malnoy et al. successfully transferred RNP into protoplasts from grapes and apples, and the mutation efficiency was as high as 6.9% [19]. To date, several investigations have reported the application of CRISPR-Cas9 gene editing technology in bananas [20–23], yet transgenic plants containing T-DNA were generated in each of these cases of CRISPR/Cas9 gene editing, and a DNA-free genome editing method has not been developed in banana. In this study, a PEG-mediated transformation system was established, which provided an effective method for the detection of gRNA activity. This system was used to successfully deliver RNPs into banana protoplasts, and the RNP system was detected to be working through deep amplicon sequencing, laying a good foundation for the further study of banana genome editing.

## Results

### Establishment of a PEG-mediated protoplast transformation system in banana

**Fig. 1:**
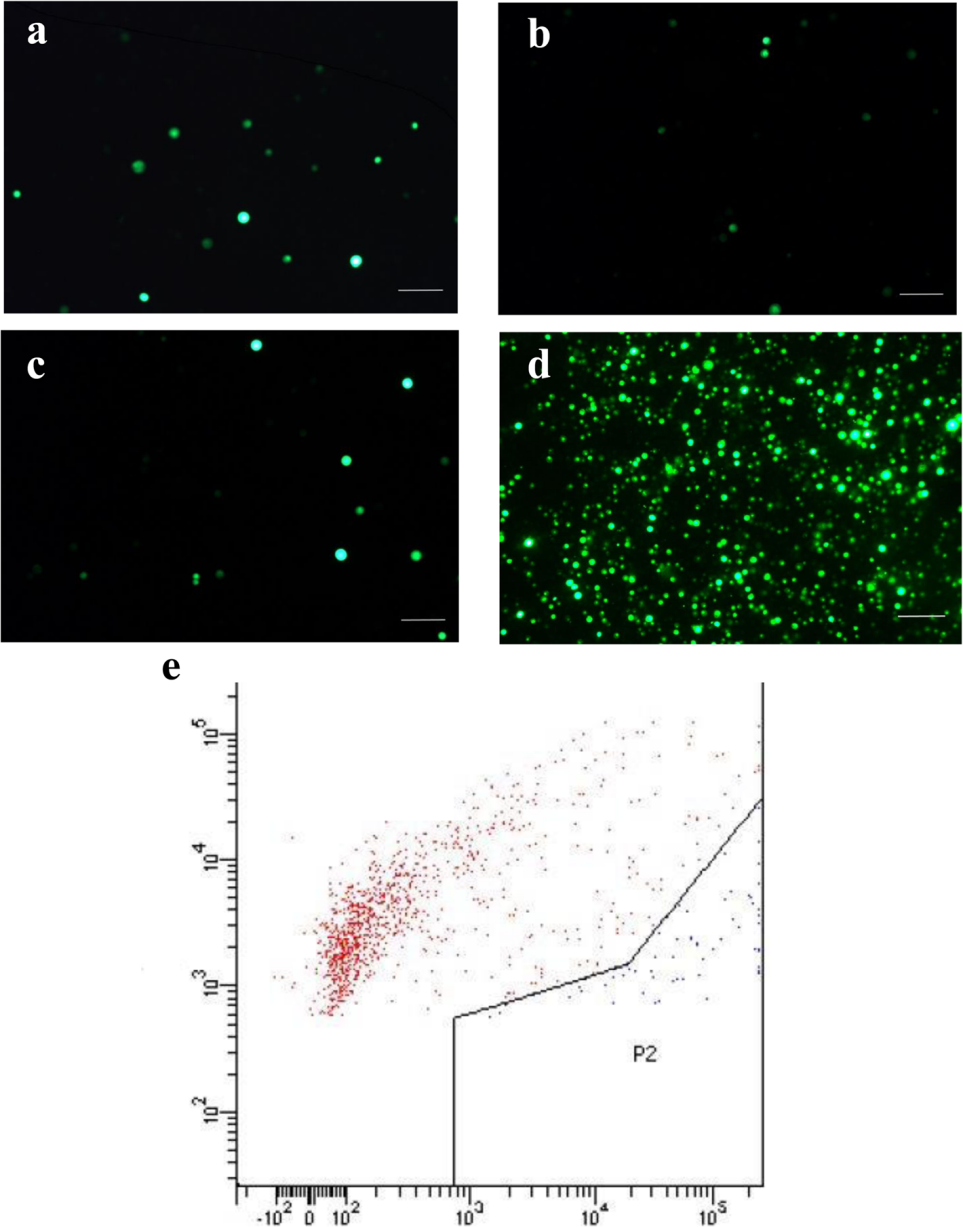
Detection of GFP fluorescence in transformed protoplasts by fluorescence microscope. pUbi-GFP plasmid was used for protoplast transformation under different transformation conditions: 40% PEG and 20 min incubation (**a**), 40% PEG and 30 min incubation (**b**), 50% PEG and 20 min incubation (**c**), 50% PEG and 30 min incubation (**d**). The protoplasts from 50% PEG with 30 min mixing were examined by flow cytometry (**e**). All pictures were taken after 5 days incubation in darkness. Scale bars are 75 μm

**Fig. 2:**
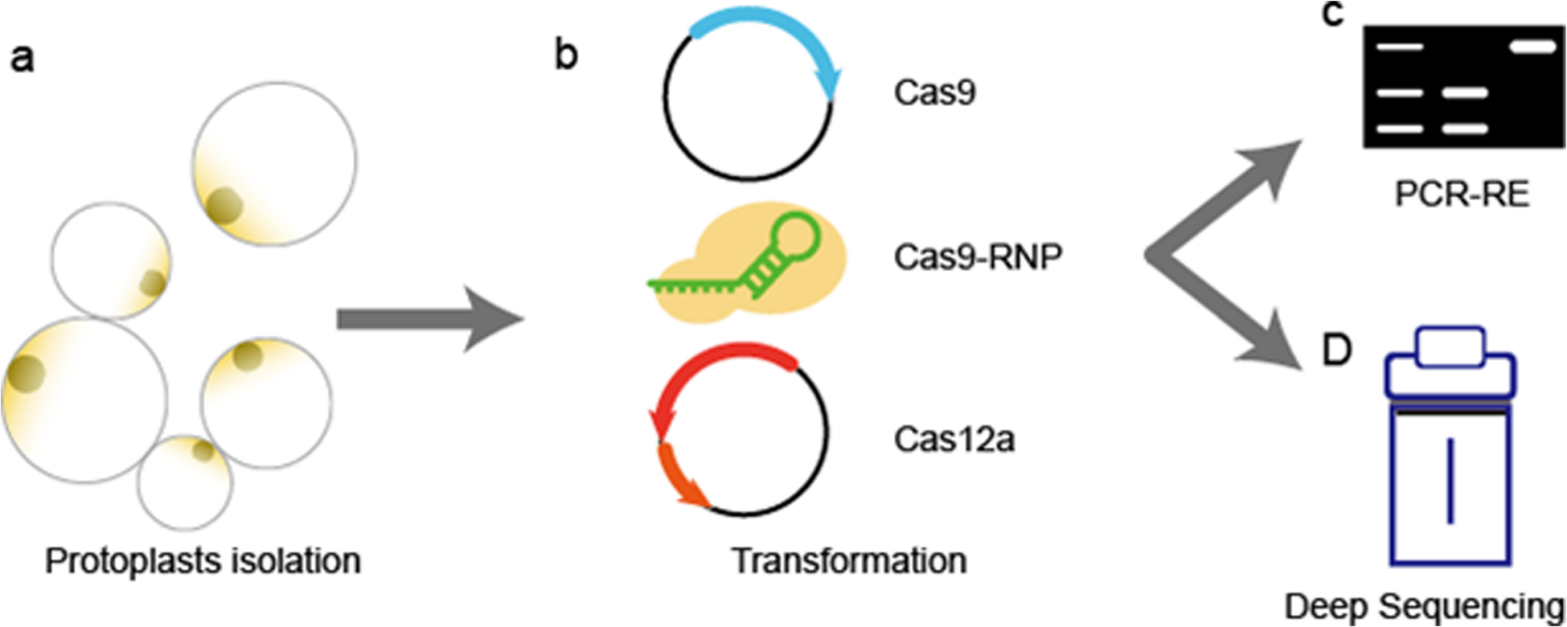
Experimental design and workflow of the transient genome editing system in banana protoplasts. The banana protoplasts were isolated from embryogenic cell suspension of Cavendish banana (AAA) (**a**), and then banana protoplasts were transformed by plasmids or RNPs through the PEG-mediated method (**b**). The genomic DNA of protoplasts was extracted 4 or 5 days after transformation. The mutation efficiency was measured by PCR-RE (**c**) or deep amplicon sequencing (**d**)

To establish a PEG-mediated banana protoplast transformation system, we optimized the transformation method based on transformation protocols for rice and wheat. The rice and wheat protocols are quite effective in rice and wheat protoplast transformation, and transformation efficiency reaches 58.4 and 64.5%, respectively, confirmed by flow cytometry detection [24]. The transformation efficiency of this protocol in banana was much lower than for rice or wheat; after 5-day incubation in darkness only a few GFP fluorescent spots appeared (Fig. 1a). Subsequently, the PEG concentration and incubation time were optimized in banana transformation with the pUbi-GFP plasmid based on rice and wheat protocols. The highest transformation rate was observed when PEG concentration was increased to 50% and induction time was as high as 30 min (Fig. 1d). The transformation efficiency was 5.6%, determined by flow cytometry detection (Fig. 1e). To determine the editing efficiency of different editing methods in banana, CRISPR/Cas9-*PDS* plasmids, CRISPR/Cas12a-*PDS* plasmids and a CRISPR/Cas9 RNP-*PDS* complex (RNPs) were examined using the optimized protoplast transformation protocol following the flow chart shown in Fig. 2.

### PEG-mediated PCR-RE assay of gene editing in banana protoplast through plasmid DNA transformation

**Fig. 3:**
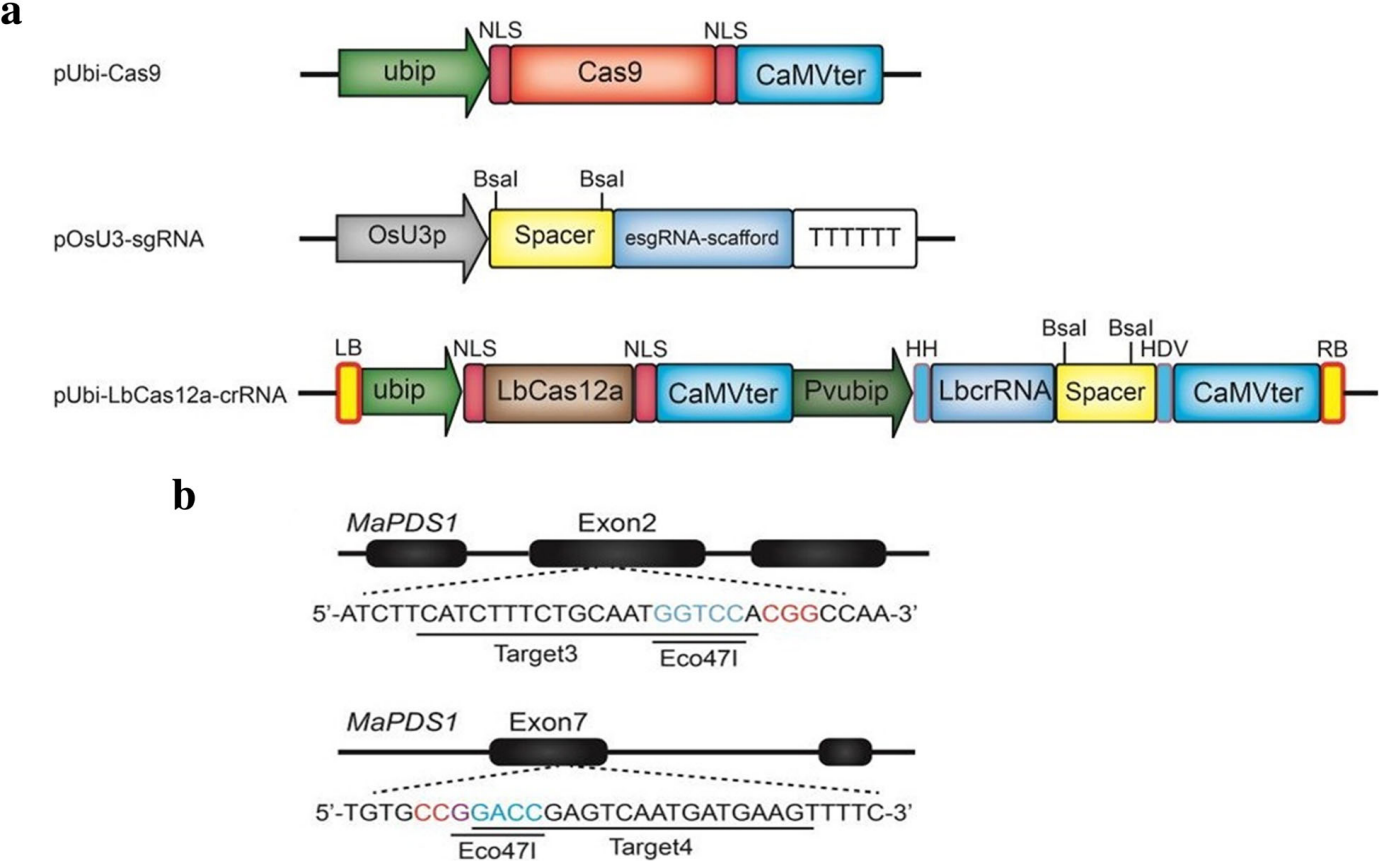
Schematic representation of the vectors and targets implemented in this study. Diagrams of pUbi*-Cas9*, sgRNA plasmid and Cas12a vector. Ubip, ubiquitin promoter from *Zea mays*. Pvubip, ubiquitin promoter from *Phaseolus vulgaris*. CaMVter, termination sequence of cauliflower mosaic virus (**a**). Details of two targets on the *PDS* gene in banana. The positions of target 3 and target 4 on the gene and the endonuclease sites are illustrated (**b**). Red letters indicate PAM sequence

**Fig. 4:**
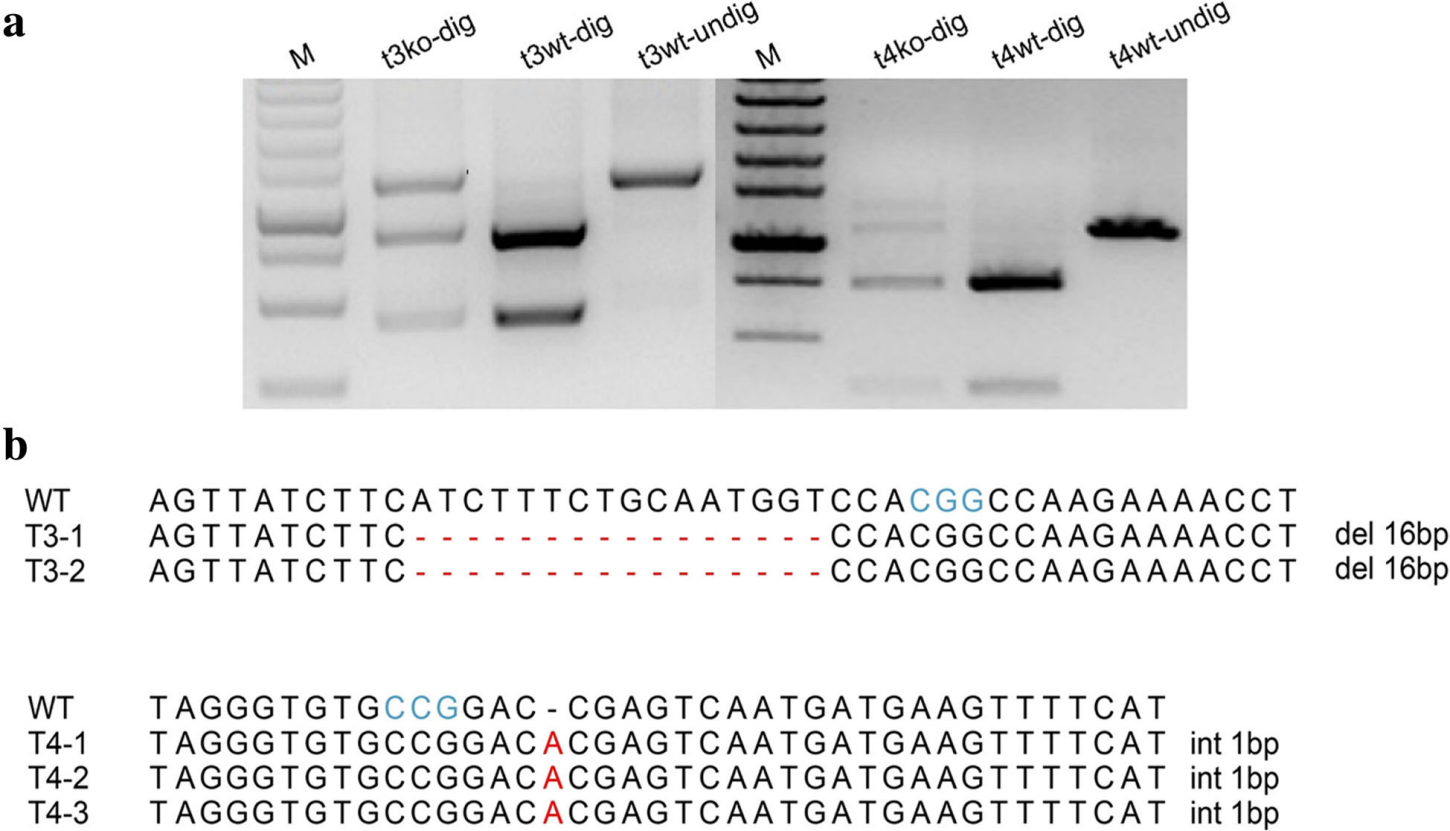
PCR-RE assay and Sanger sequencing of single clones after transferring plasmid DNA into banana protoplasts. The PCR-amplified fragment of edited target 3 (t3ko) or its wild type (t3wt) and edited target 4 (t4ko) or its wild type (t4wt) were digested by *Eco*47I (dig) or were not digested (undig). Gene edited fragments were amplified with the DNA from transformed protoplasts, while wild-type target fragments were amplified with the DNA from untransformed protoplasts. M indicates DNA marker (**a**). Sanger sequencing of single clones of undigested fragments from Fig. 4a (**b**). The blue letters indicate the PAM sequence; red letters signify mutations with base insertions; a red ‘-‘means a mutation with a base deletion

To explore whether our protoplast transformation system can be applied to genome editing by transferring plasmid DNAs, we designed 9 sgRNAs, of which two sgRNAs target the 2nd (target 3: OsU3p-PDSt3) and 7th exon (target 4: OsU3p-PDSt4) of the banana *PDS* gene, respectively. These two sgRNAs both contain recognition sites for *Eco*47I that can be used for PCR-RE assays (Fig. 3). Each of these 9 guided RNAs were fused with an enhanced scaffold RNA (Fig. 3a). These plasmid DNAs and the Cas9 plasmid were transformed into the banana protoplasts using the abovementioned method. DNA isolated from resulting transformed and nontransformed (control) protoplasts were used for PCR-RE assays and sequencing analysis. PCR-RE results showed that PCR product from the control sample was completely digested into two bands (t3wt-dig, t4wt-dig), but PCR products from plasmid-transformed samples were only partially digested under the same conditions (t3ko-dig, t4ko-dig), suggesting that the restriction enzyme site was mutated by the gene editing cassette (Fig. 4a). To further characterize the mutation type (i.e., insertion, deletion, etc.) created by Cas9, the PCR fragments undigested by *Eco*47I were recovered and cloned into T-blunt vectors for Sanger sequencing. Ten single clones for OsU3p- PDSt3 and three single clones for OsU3p- PDSt4 were selected for Sanger sequencing. The results indicated that a 16-bp deletion was present in two cloned fragments of OsU3p- PDSt3, and all three cloned fragments of OsU3p- PDSt4 exhibited a 1-bp insertion. The insertion and the deletion started at the fourth nucleotide from the PAM sequence (Fig. 4b). We also designed 11 sgRNAs of banana *PDS* gene for LbCas12a, but we did not check the results by PCR-RE assay.

### PCR-RE assay of gene editing in banana protoplast transformed with RNP

**Fig. 5:**
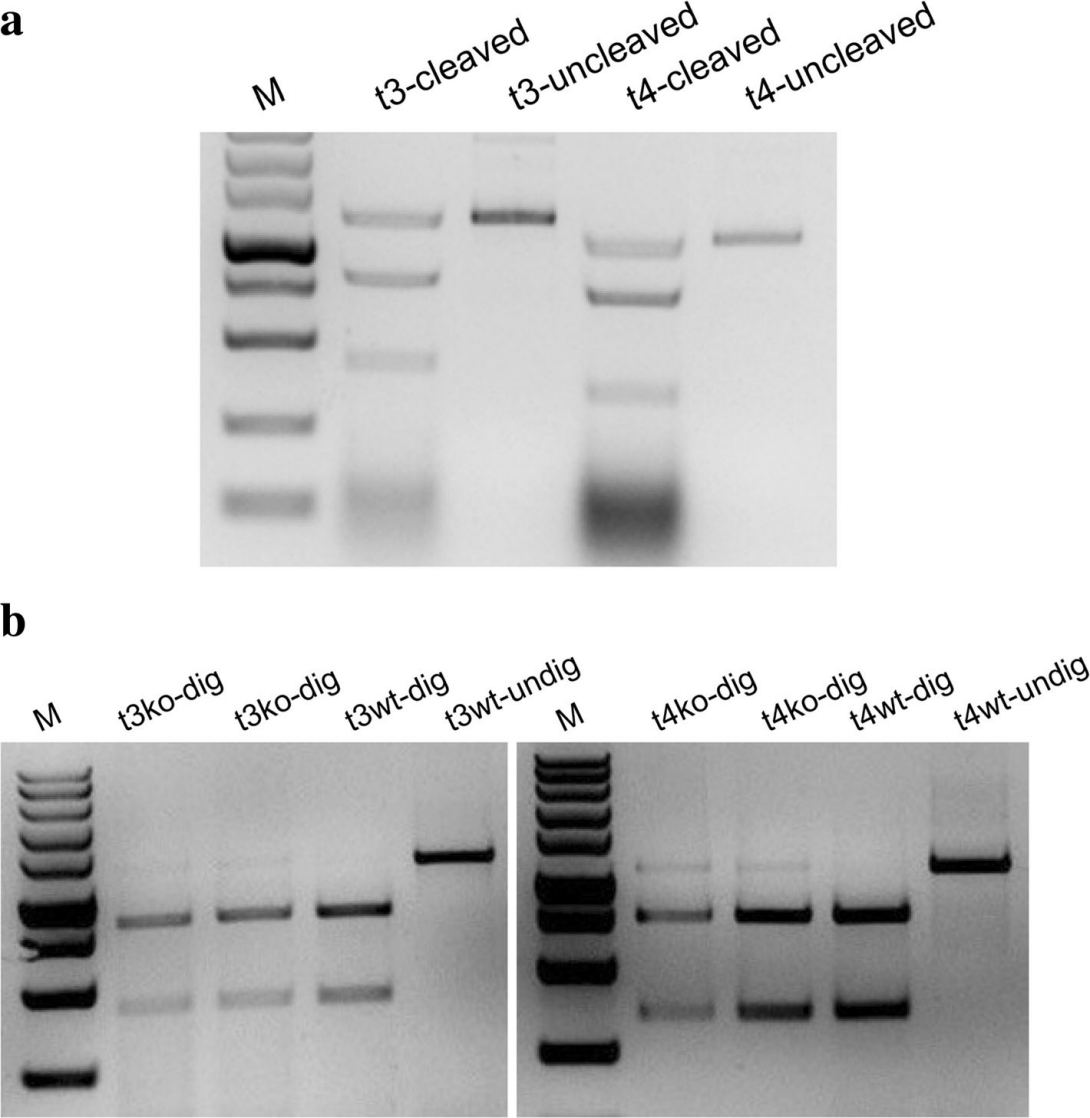
RNP cleavage test in vitro and PCR-RE test after protoplast transformation. RNP cleavage test in vitro; t3-cleaved and t4-cleaved were used to cut the target 3 and target 4 PCR sequences by RNPs, while t3-uncleaved, as a control, was the sequence of target 3 PCR, and t4-uncleaved was the target 4 PCR sequence (**a**). ko-dig refers to *Eco*471 enzyme digestion of PCR amplification target sequences after RNP transformation of protoplasts; wt-dig refers to *Eco*471 enzyme digestion of target sequences of PCR amplification after blank transformation of protoplasts; wt-undig refers to PCR amplification after blank transformation of protoplasts, as a control. M indicates DNA marker (**b**)

To detect whether target sites mutated after RNP complexes were delivered into protoplasts, we transferred the complex of purified Cas9 proteins with targeting sgRNAs into the banana protoplasts according to the method reported by Liang [25, 26]. We first tested whether RNPs could efficiently edit the targets in vitro prior to using them to transform protoplasts. As results show in Fig. 5a, RNPs were able to cleave the targets contained in the PCR product into two fragments. We next isolated DNA from the transformed and nontransformed protoplasts for PCR amplification. Amplified fragments containing target sequences were subjected to digestion with *Eco*47I. The results showed that a substantial quantity of amplified fragment could not be digested in the RNP-transformed sample, whereas amplicon from the control sample could be completely digested, suggesting the disruption of the *Eco*47 I recognition site by RNP in the RNP-transformed sample (Fig. 5 b).

### Deep amplicon sequencing of DNA transformation mediated by PEG in banana protoplasts

Cavendish banana protoplasts were separately transformed with pUbi - Cas9 plasmid mixed with each of 9 sgRNA plasmids targeting *PDS* and each of 11 Cas12a - PDS plasmids (Cas12a - PDSt1 to Cas12a - PDSt11). To examine the editing efficiency, genomic DNAs were isolated from protoplasts transformed with each sgRNA plus Cas9 or LbCas12a plasmid for deep amplicon sequencing. The sequencing results showed that 5 target sites (PDSt4, PDSt6, PDSt7, PDSt8 and PDSt9) were edited in the protoplasts transformed with sgRNAs and Cas9 plasmids. Among these edited target sites, the target site PDSt8 exhibited the highest editing efficiency of 1.04%, and 320 inserts and 800 deletions out of 107,568 sequencing reads for target site PDSt8 were observed. The lowest editing efficiency of 0.18% occurred for the target site PDSt6. Mutations were observed out of 80,384 sequencing reads, and 147 sequencing reads contained various deletions (Additional file 1:Table S1).

For the LbCas12a system, four target sites (Cas12a- PDSt1, Cas12a- PDSt7, Cas12a- PDSt9 and Cas12a- PDSt10) were successfully edited. The highest editing was observed for the target site Cas12a- PDSt9 with an efficiency of 0.39%, and deletion-type editing was found in 114 out of 29,008 sequencing reads for Cas12a- PDSt9. In contrast, only 78 (3 insertions and 75 deletions) of 52,965 sequencing reads contained mutations created in the target site Cas12a-PDSt7, resulting in the lowest editing efficiency (0.15%) at this target site (Additional file 2:Table S2).

### Deep amplicon sequencing of 9 complexes of Cas9 and sgRNAs for PEG-mediated transformation of banana protoplasts

Cas9 proteins combined separately with 9 sgRNAs were transformed into banana protoplasts. To examine the editing efficiency of transforming RNPs, we also performed deep amplicon sequencing of genomic DNA isolated from the protoplasts transformed with the 9 complexes of Cas9 and sgRNAs targeting 9 different *PDS* sites. We have detected editing by this RNP system at five target sites, including PDS-sgRNAt2, PDS -sgRNAt4, PDS -sgRNAt6, PDS -sgRNAt7 and PDS -sgRNAt9. The target site PDS -sgRNAt9 had the lowest editing efficiency (0.19%), including 166 insertion reads and 26 deletion reads out of 102,866 reads. The target site PDS -sgRNAt6 exhibited the highest editing efficiency (0.92%), including 169 insertion reads and 1104 deletion reads out of 138,085 reads (Additional file 3:Table S3).

### Off-target detection

To analyze the off-target effect of CRISPR/Cas9-*PDS* plasmids and the CRISPR/Cas9 RNP-*PDS* complex (RNPs) gene editing system, a potential off-target site (AGCTTCGTGTACCGCAGTAGTGG), GSMUA_Achr6G21680_001, was predicted via the CRISPR-P 2.0 website. There are four base mismatches between the off-target site and the sgRNA sequence. Through deep amplicon sequencing, only one site was detected in the CRISPR/Cas9-*PDS* and RNPs gene editing system, and the off-target efficiency was 0.01%. (Additional file 4:Table S4, Additional file 5:Table S5, Additional file 6:Table S6).

### Discussion

Banana is a kind of tropical and subtropical monocotyledonous perennial herbaceous plant. Most cultivated varieties are triploid, with high fertility. As there is no seed in edible bananas, it is very difficult to achieve fine varieties with good quality and strong disease-resistance through traditional crossbreeding cultivation. However, there is a certain blindness and longer development cycle requirement with breeding new varieties by mutation breeding and mutant screening. For stable CRISPR/Cas9 gene editing by *Agrobacterium*-mediated transformation in major banana cultivars, the sterility of pollen makes it difficult to remove the exogenous integrated DNA by crossing as is done with diploid plants. Due to the absence of exogenous DNA integrated into the genome in gene editing with CRISPR/Cas9 RNP complexes, this approach provides an effective option for banana molecular breeding by gene editing.

Woo et al. (2015) reported that CRISPR/Cas9 RNP complexes can be delivered into the protoplasts of *Arabidopsis thaliana*, tobacco, lettuce and rice for gene editing, and the mutation efficiency of the target in tobacco was as high as 44% [18]. Malnoy et al.(2016) delivered RNPs into grape and apple protoplasts by PEG, and the mutation efficiency in apples reached 6.9%, as measured by deep amplicon sequencing [19]. In 2016, Svitashev et al. directly delivered an RNP complex into immature embryos of corn by biolistic bombardment and achieved targeted gene editing with 0.69% mutation efficiency [27]. Similarly, Liang et al. (2017) developed an efficient DNA-free genome editing method in bread wheat using Cas9 RNP complexes by particle bombardment with 0.56% mutation efficiency [25]. These results indicate that the mutation efficiency of RNP complexes using PEG-mediated protoplast transformation is much higher than that using biolistic bombardment.

**Fig. 6:**
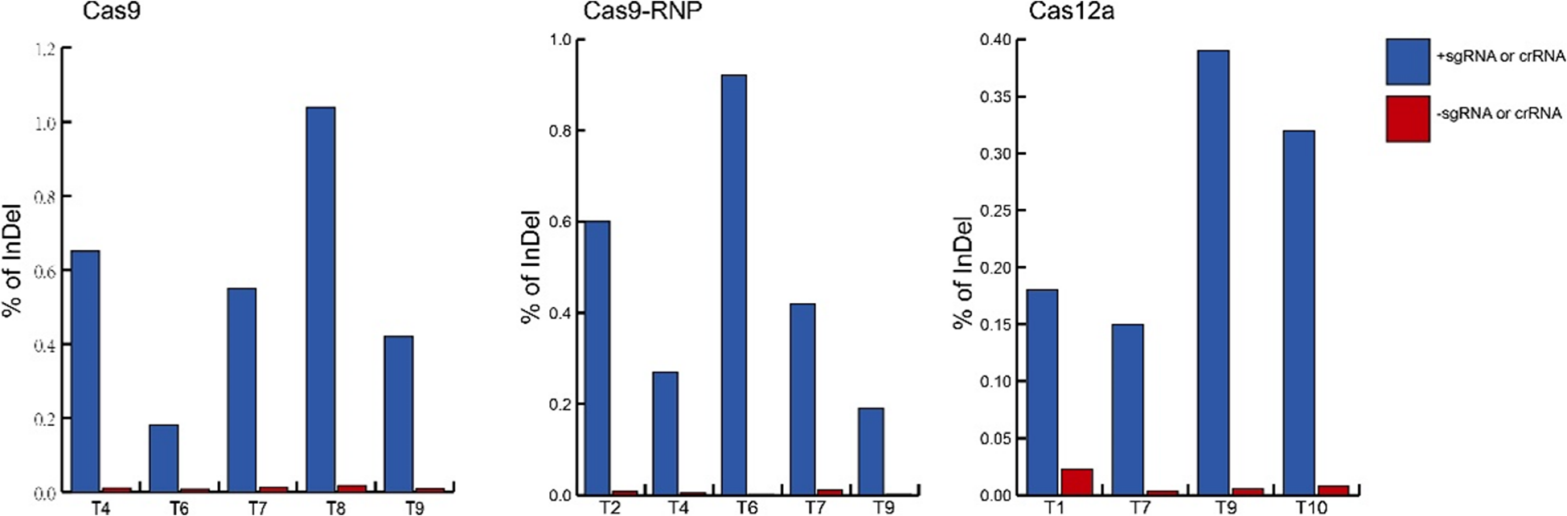
Frequencies of indels introduced by Cas9, RNP and Cas12a genome editing systems

Since CRSPR/Cas9 gene editing technology was first reported in 2013, it has been widely used and rapidly developed. A protoplast transient transformation system can effectively detect the activity of target gene sites, providing an effective means for screening gRNA and is a very good auxiliary tool for the application of CRSPR/Cas9 gene editing technology. In this study, banana protoplast systems were transformed with Cas9 and OsU3p- PDS, RNPs, and Cas12a-PDS. Through deep amplicon sequencing, mutation efficiency of the Cas9 system was found to be greater than that of an RNP at the same target; for example Cas9 and OsU3p- PDSt4 (0.65%) > PDSt4-RNP (0.27%), and Cas9 and OsU3p- PDSt7 (0.55%) > PDSt7-RNP (0.42%), Cas9 and OsU3p- PDSt9 (0.42%) > PDSt9-RNP (0.19%). Among 9 target sites, the highest editing efficiency of the detected Cas9 system was achieved by Cas9 and OsU3p - PDSt8, with a mutation efficiency of 1.04%; the highest mutation efficiency of a detected RNP system was for PDSt6-RNP, with a mutation efficiency of 0.92%; the highest editing efficiency of a detected Cas12a system was for Cas12a- PDSt9, with a mutation efficiency of 0.39%, mostly consistent with the predicted results (Fig. 6).

Recently, CRISPR/Cas9 technology has been applied to knock out the *PDS* gene in banana to achieve an albino mutant [20–22] and to disrupt the *MaGA20ox2* gene to obtain semidwarf banana material [23]. However, there have been no reports about transformation of banana protoplasts with RNPs or the delivery of RNPs into embryogenic cell suspension (ECS) of banana. Although it has been reported that regenerated plants can be obtained by nursing culture of banana protoplasm [28, 29], this method displays low regeneration rate, poor reproducibility, and difficult regeneration. Therefore, further research on the improvement of plant regeneration efficiency from Cas9 RNP complex transformed protoplasts is very important for the application of this technology in banana breeding. The mutation efficiency from transformation of protoplasts with RNPs is much higher than that from delivery of RNP into ECS by biolistic bombardment. There is a high potential that regeneration plants free of exogenous DNA can be achieved from banana protoplasts transformed with RNP by the PEG-mediated method, as long as an increase in banana protoplast regeneration efficiency can be accomplished. This proposal has vital significance in genetic improvement of banana and generation of new non-transgenic germplasms.

## Conclusion

In this study, we optimized and established a banana protoplast transformation method base on protocols for rice and wheat, which is useful for gRNA activity validation. In addition, CRISPR/Cas9, CRISPR/Cas12a and a Cas9 RNP complex can successfully edit endogenous genes via an optimized protocol. The efficiency of the CRISPR/Cas9 system was greater than for the other two systems. In addition, delivering the Cas9-RNP complex into protoplasts using the PEG-mediated method shows apparent advantages compared to biolistic bombardment of ECS, and further research on plant regeneration from protoplasts is critical for successfully establishing a DNA-free gene editing system in banana.

## Methods

### Plant material

Plant material used in this study was Cavendish Banana (*Musa* spp. Cavendish; AAA Group cv. ‘Baxi’); the ‘Baxi’ banana is one banana cultivar that has been grown in China for many years and is one of the main banana cultivars in China. We obtained male flower buds of ‘Baxi’ banana from the Institute of Fruit Tree Research, Guangdong Academy of Agricultural Sciences, Guangzhou, P. R. of China. ECS was induced by our laboratory in the Institute of Fruit Tree Research, Guangdong Academy of Agricultural Sciences.

### Banana protoplast preparation

Small and evenly distributed subculture of ECS, cultured for approximately 10 days, was selected; the M2 medium was removed, 10 ml of enzymatic hydrolysate (3.0% cellulose R^− 10^, 1% segregation enzyme R-10, 0.2% pectinase Y-23, 15.2 g/L KCl, 7.8 g/L CaCl_2_, 100 mg/L MES, 10% mannitol, pH 5.7) was added; and the cells were incubated in a shaking table at 50 rpm/min for 6–8 h. The yield of protoplasts was observed by microscope. If the enzymatic hydrolysis was sufficient, the hydrolysate was diluted with 10 ml W5 solution and shaken for 10 s to separate the protoplasts. A 75-μm membrane was used to filter the enzymatic solution into a round-bottom centrifugal tube. Centrifugation was performed at 100 g for 3 min, and the supernatant was removed by pipette. Protoplasts were suspended in 15 ml W5 solution and incubated on ice for 30 min, and the supernatant was discarded.

### gRNA design and vector construction

A total of 9 vectors targeting the *PDS* gene in banana were designed by SnapGene software. The OsU3p vector was digested by Bsa1, and the fragments were recovered from agarose gel. The serial joint primers (Additional file 7:Table S7) were linked to the recycled OsU3p vector by T4 ligase to generate OsU3p- PDSt1 to OsU3p- PDSt9 and then used to transform *E. coli DH5α* competent cells. After overnight culture at 37 °C, single colonies verified by sequencing were inoculated in LB liquid medium with ampicillin. After overnight culture at 37 °C 220 rpm, plasmids were extracted.

### CRISPR/Cas12a vector construction

A total of 11 vectors targeting the *PDS* gene in banana were designed by SnapGene software. The Cas12a vector was digested by Bsa1, and the fragments were recovered from agarose gel. The serial joint primers (Additional file 8:Table S8) were linked to the recycled Cas12a vector by T4 ligase to generate Cas12a- PDSt1 to Cas12a- PDSt11 and then used to transform *E. coli DH5α* competent cells. After overnight culture at 37 °C, single colonies verified by sequencing were inoculated in LB liquid medium with kanamycin. After overnight culture at 37 °C and 220 RPM, plasmids were extracted.

### Transcription of sgRNAs in vitro

Specific primers of target sites (Additional file 9:Table S9) were designed, using OsU3p- PDSt1 to OsU3p-PDSt9 plasmids as templates, amplified by the high-fidelity enzyme FastPfu and purified using the EasyPure PCR Kit. Transcription of purified PCR products in vitro was performed by the NEB HiScribe™ T7 in vitro Transcription Kit. In vitro transcription products were purified by the TIANGEN RNA Purification Kit.

### Protoplast transformation with plasmid or Cas9 RNP complex

The protoplast concentration was adjusted to 2 × 10^6^–2 × 10^7^ with MMG, and protoplasts were incubated on ice. 20 μg plasmids were added to a 2-ml centrifuge tube and precipitated to the bottom of the tube by centrifugation. 200 μl protoplasts were added into the tube, and the contents were lightly mixed. 250 μl 50% PEG 4000 was added and induced transformation for 30 min in darkness. Addition of 900 μl W5 solution stopped transfection. The sample was centrifuged at 100 g for 3 min, and supernatant was discarded. The protoplasts were resuspended with 1 ml W5 and cultured in darkness at 26–28 °C.

According to the method described above, 10 μg pUbi-Cas9 plasmid was mixed separately with 10 μg of plasmids OsU3p- PDSt1 to OsU3p- PDSt9, 20 μg of plasmids Cas12a- PDSt1 to Cas12a- PDSt11, Cas9 protein (20 μg) and sgRNA (20 μg). Banana protoplasts were separately transformed by each of the prepared samples using the PEG method and then dark-cultured. The pUbi-GFP plasmid was used for transformation as a control.

### PCR-RE test and sanger sequencing of single clones

After DNA and RNP transformation, the target sites with specific endonuclease sites were selected for PCR - RE tests. Genomic DNA of protoplasts was extracted. Sequences with lengths of approximately 1000 bp containing target sites were amplified by PFU enzyme. The specific PCR products were digested by Eco471 at 37 °C for 2 h and analyzed on 2% agarose gel by electrophoresis. To determine whether there were specific fragments with base mutations, the specific fragments were recovered after electrophoresis, connected to the T-blunt vector, used to transform *E. coli* DH5α competent cells and then selected for Sanger sequencing of single clones.

### RNP cleavage in vitro

DNA fragments containing target 3 and target 4 were amplified by PCR, purified using the EasyPure PCR Purification Kit, and eluted by RNase-free water. The cleavage reaction system in vitro was as follows: Cas9 protein (1 μg), sgRNA (1 μg), target fragment (100 ng), 10 × Cas9 reaction buffer (20 mM HEPES, pH 7.5, 150 mM KCl, 10 mM MgCl_2_, 0.5 mM DTT) 2 μl, RNase-free water up to total volume of 20 μl. Samples were incubated at 37 °C for 1 h and then at 65 °C for 10 min. Finally, samples were tested by electrophoresis on 2% agarose gel.

### Deep amplicon sequencing

Banana protoplasts were transformed with prepared samples of DNA or RNP. After 4–5 days dark culture, the banana protoplasts were collected by centrifugation at 12000 RPM, and then genomic DNA was extracted with the TIANGEN DNA Extraction Kit. Deep amplicon sequencing primers (Additional file 10:Table S10, Additional file 11:Table S11) were designed, and nested PCR was performed to amplify fragments with approximate lengths of 200 bp. After gel purification, samples were sent for deep amplicon sequencing by Shanghai Shenggong Biology Co., Ltd., to determine whether there were base mutations in the target sequences and the types of mutations.

## Supplementary information

**Additional file 1: Table S1.** The results of deep amplicon sequencing of Cas9 system.

**Additional file 2: Table S2.** The results of deep amplicon sequencing of Cas12a system.

**Additional file 3: Table S3.** The results of deep amplicon sequencing of RNP system.

**Additional file 4: Table S4.** Off-target effects of Cas9 system.

**Additional file 5: Table S5.** Off-target effects of RNP system.

**Additional file 6: Table S6.** Primer pairs for off-target detection.

**Additional file 7: Table S7.** Primer pairs used to construct OsU3p-PDS.

**Additional file 8: Table S8.** Primer pairs used to construct Cas12a-PDS.

**Additional file 9: Table S9.** Primer pairs used for in vitro transcription of sgRNA.

**Additional file 10: Table S10.** Primer pairs used for capture-sequencing of Cas9 and RNP system.

**Additional file 11: Table S11.** Primer pairs used for capture-sequencing of Cas12a system.

## Data Availability

Deep amplicon sequencing data are available under BioProject IDs PRJNA637446 (https://www.ncbi.nlm.nih.gov/sra/PRJNA637446), PRJNA637703 (https://www.ncbi.nlm.nih.gov/sra/PRJNA637703) and PRJNA637699 (https://www.ncbi.nlm.nih.gov/sra/PRJNA637699).

## Abbreviations

PEG: Polyethylene glycol
CRISPR: Clustered regularly interspaced short palindromic repeats
RNP: Ribonucleoprotein
PCR-RE: PCR-Restriction Enzyme
PDS: Phytoene dehydrogenase
GFP: Green fluorescent protein
ECS: Embryogenic cell suspension
sgRNA: Single-guide RNA
