# Supplementary material for: Establishment of a PEG-mediated protoplast transformation system based on DNA and CRISPR/Cas9 ribonucleoprotein complexes for banana
Source: BMC Plant Biol. 2020 Sep 15;20:425. doi: 10.1186/s12870-020-02609-8 (PMC7493974; doi:10.1186/s12870-020-02609-8)
Supplement: Supplementary file 1 — Additional file 1: Table S1. The results of deep amplicon sequencing of Cas9 system. [file 12870_2020_2609_MOESM1_ESM.docx]

**Additional file 1：Table S1. The results of deep amplicon sequencing of Cas9 system**

| **Targets** | **Reads** | **inserts** | **Deletions** | **Editing efficiency** |
| --- | --- | --- | --- | --- |
| **MAPDSt1_KO** | **50589** | **1** | **3** | **0.01%** |
| **MAPDSt1_WT** | **16752** | **1** | **3** | **0.02%** |
| **MAPDSt2_KO** | **83480** | **2** | **4** | **0.01%** |
| **MAPDSt2_WT** | **29223** | **0** | **12** | **0.04%** |
| **MAPDSt3_KO** | **115558** | **36** | **5** | **0.04%** |
| **MAPDSt3_WT** | **28757** | **0** | **10** | **0.03%** |
| **MAPDSt4_KO** | **119303** | **444** | **337** | **0.65%** |
| **MAPDSt4_WT** | **85985** | **2** | **4** | **0.01%** |
| **MAPDSt5_KO** | **22** | **0** | **0** | **0.00%** |
| **MAPDSt5_WT** | **14** | **1** | **0** | **7.14%** |
| **MAPDSt6_KO** | **80384** | **0** | **147** | **0.18%** |
| **MAPDSt6_WT** | **32119** | **0** | **4** | **0.01%** |
| **MAPDSt7_KO** | **120862** | **127** | **539** | **0.55%** |
| **MAPDSt7_WT** | **55854** | **1** | **16** | **0.03%** |
| **MAPDSt8_KO** | **107568** | **320** | **800** | **1.04%** |
| **MAPDSt8_WT** | **38919** | **0** | **5** | **0.01%** |
| **MAPDSt9_KO** | **76852** | **204** | **115** | **0.42%** |
| **MAPDSt9_WT** | **14379** | **0** | **1** | **0.01%** |
