# Supplementary material for: Establishment of a PEG-mediated protoplast transformation system based on DNA and CRISPR/Cas9 ribonucleoprotein complexes for banana
Source: BMC Plant Biol. 2020 Sep 15;20:425. doi: 10.1186/s12870-020-02609-8 (PMC7493974; doi:10.1186/s12870-020-02609-8)
Supplement: Supplementary file 2 — Additional file 2: Table S2. The results of deep amplicon sequencing of Cas12a system. [file 12870_2020_2609_MOESM2_ESM.docx]

**Additional file 2：Table S2. The results of deep amplicon sequencing of Cas12a system**

| **Targets** | **Reads** | **inserts** | **Deletions** | **Editing efficiency** |
| --- | --- | --- | --- | --- |
| **MACPF1_t1** | **44984** | **1** | **81** | **0.18%** |
| **MACPF1t1_WT** | **16428** | **11** | **1** | **0.07%** |
| **MACPF1_t2** | **21878** | **1** | **15** | **0.07%** |
| **MACPF1t2_WT** | **8813** | **0** | **2** | **0.02%** |
| **MACPF1_t3** | **26805** | **0** | **25** | **0.09%** |
| **MACPF1t3_WT** | **8850** | **0** | **1** | **0.01%** |
| **MACPF1_t4** | **216728** | **1** | **18** | **0.01%** |
| **MACPF1t4_WT** | **130179** | **1** | **7** | **0.01%** |
| **MACPF1_t5** | **0** | **0** | **0** | **0.00%** |
| **MACPF1t5_WT** | **0** | **0** | **0** | **0.00%** |
| **MACPF1_t6** | **62** | **43** | **9** | **83.87%** |
| **MACPF1t6_WT** | **18** | **16** | **0** | **88.89%** |
| **MACPF1_t7** | **52965** | **3** | **75** | **0.15%** |
| **MACPF1t7_WT** | **26512** | **0** | **1** | **0.00%** |
| **MACPF1_t8** | **56866** | **1** | **52** | **0.09%** |
| **MACPF1t8_WT** | **26399** | **0** | **2** | **0.01%** |
| **MACPF1_t9** | **29008** | **0** | **114** | **0.39%** |
| **MACPF1t9_WT** | **36703** | **1** | **1** | **0.01%** |
| **MACPF1_t10** | **27728** | **1** | **87** | **0.32%** |
| **MACPF1t10_WT** | **36885** | **0** | **3** | **0.01%** |
| **MACPF1_t11** | **23931** | **2** | **0** | **0.01%** |
| **MACPF1t11_WT** | **36909** | **1** | **1** | **0.01%** |
