# Supplementary material for: Establishment of a PEG-mediated protoplast transformation system based on DNA and CRISPR/Cas9 ribonucleoprotein complexes for banana
Source: BMC Plant Biol. 2020 Sep 15;20:425. doi: 10.1186/s12870-020-02609-8 (PMC7493974; doi:10.1186/s12870-020-02609-8)
Supplement: Supplementary file 3 — Additional file 3: Table S3. The results of deep amplicon sequencing of RNP system. [file 12870_2020_2609_MOESM3_ESM.docx]

**Additional file 3：Table S3. The results of deep amplicon sequencing of RNP system**

| **Targets** | **Reads** | **inserts** | **Deletions** | **Editing efficiency** |
| --- | --- | --- | --- | --- |
| **MARNPPDS_t1** | **81613** | **45** | **36** | **0.10%** |
| **MARNPPDSt1_WT** | **76687** | **0** | **11** | **0.01%** |
| **MARNPPDS_t2** | **110993** | **395** | **267** | **0.60%** |
| **MARNPPDSt2_WT** | **116895** | **0** | **6** | **0.01%** |
| **MARNPPDS_t3** | **226711** | **70** | **30** | **0.04%** |
| **MARNPPDSt3_WT** | **162757** | **0** | **14** | **0.01%** |
| **MARNPPDS_t4** | **288822** | **343** | **442** | **0.27%** |
| **MARNPPDSt4_WT** | **186542** | **0** | **6** | **0.00%** |
| **MARNPPDS_t5** | **16** | **0** | **0** | **0.00%** |
| **MARNPPDSt5_WT** | **13** | **0** | **0** | **0.00%** |
| **MARNPPDS_t6** | **138085** | **169** | **1104** | **0.92%** |
| **MARNPPDSt6_WT** | **141210** | **0** | **22** | **0.02%** |
| **MARNPPDS_t7** | **211619** | **591** | **305** | **0.42%** |
| **MARNPPDSt7_WT** | **183742** | **0** | **14** | **0.01%** |
| **MARNPPDS_t8** | **196805** | **23** | **110** | **0.07%** |
| **MARNPPDSt8_WT** | **165821** | **0** | **10** | **0.01%** |
| **MARNPPDS_t9** | **102866** | **166** | **26** | **0.19%** |
| **MARNPPDSt9_WT** | **119080** | **0** | **3** | **0.00%** |
