# Supplementary material for: Establishment of a PEG-mediated protoplast transformation system based on DNA and CRISPR/Cas9 ribonucleoprotein complexes for banana
Source: BMC Plant Biol. 2020 Sep 15;20:425. doi: 10.1186/s12870-020-02609-8 (PMC7493974; doi:10.1186/s12870-020-02609-8)
Supplement: Supplementary file 4 — Additional file 4: Table S4. Off-target effects of Cas9 system. [file 12870_2020_2609_MOESM4_ESM.docx]

**Additional file 4：Table S4. Off-target effects of Cas9 system**

| **Targets** | **Reads** | **inserts** | **Deletions** | **Editing efficiency** |
| --- | --- | --- | --- | --- |
| **MAOFFTARGET_1** | **124266** | **1** | **2** | **0.00%** |
| **MAOFFTARGET_2** | **133552** | **3** | **2** | **0.00%** |
| **MAOFFTARGET_3** | **119751** | **0** | **4** | **0.00%** |
| **MAOFFTARGET_4** | **102579** | **0** | **3** | **0.00%** |
| **MAOFFTARGET_5** | **140420** | **0** | **10** | **0.01%** |
| **MAOFFTARGET_6** | **123859** | **0** | **2** | **0.00%** |
| **MAOFFTARGET_7** | **122724** | **0** | **2** | **0.00%** |
| **MAOFFTARGET_8** | **120746** | **0** | **3** | **0.00%** |
| **MAOFFTARGET_9** | **134415** | **1** | **0** | **0.00%** |
| **MAOFFTARGET_WT** | **119352** | **1** | **3** | **0.00%** |
