# Supplementary material for: Establishment of a PEG-mediated protoplast transformation system based on DNA and CRISPR/Cas9 ribonucleoprotein complexes for banana
Source: BMC Plant Biol. 2020 Sep 15;20:425. doi: 10.1186/s12870-020-02609-8 (PMC7493974; doi:10.1186/s12870-020-02609-8)
Supplement: Supplementary file 5 — Additional file 5: Table S5. Off-target effects of RNP system. [file 12870_2020_2609_MOESM5_ESM.docx]

**Additional file 5：Table S5. Off-target effects of RNP system**

| **Targets** | **Reads** | **inserts** | **Deletions** | **Editing efficiency** |
| --- | --- | --- | --- | --- |
| **MAOFFTARGET_1** | **206250** | **1** | **6** | **0.00%** |
| **MAOFFTARGET_2** | **283105** | **1** | **17** | **0.01%** |
| **MAOFFTARGET_3** | **253061** | **0** | **6** | **0.00%** |
| **MAOFFTARGET_4** | **205521** | **0** | **7** | **0.00%** |
| **MAOFFTARGET_5** | **236819** | **0** | **10** | **0.00%** |
| **MAOFFTARGET_6** | **226224** | **1** | **10** | **0.00%** |
| **MAOFFTARGET_7** | **257234** | **0** | **9** | **0.00%** |
| **MAOFFTARGET_8** | **230694** | **0** | **7** | **0.00%** |
| **MAOFFTARGET_9** | **211818** | **1** | **8** | **0.00%** |
| **MAOFFTARGET_WT** | **283419** | **1** | **2** | **0.00%** |
