# Supplementary material for: Establishment of a PEG-mediated protoplast transformation system based on DNA and CRISPR/Cas9 ribonucleoprotein complexes for banana
Source: BMC Plant Biol. 2020 Sep 15;20:425. doi: 10.1186/s12870-020-02609-8 (PMC7493974; doi:10.1186/s12870-020-02609-8)
Supplement: Supplementary file 6 — Additional file 6: Table S6. Primer pairs for off-target detection. [file 12870_2020_2609_MOESM6_ESM.docx]

**Additional file 6：Table S6. Primer pairs of off - tatget detection**

| **Name** | **Primer_F** | **Primer_R** |
| --- | --- | --- |
| **MA21680** | **TTCCCCTCTCCTATACGGTGTCGCCCTG** | **TGCTGAATCATTTATCTGCCTTCTTTC** |
| **ngsMA21680-1** | **CGATGTAAGCGGAAAACAGAGCCGAG** | **TGACCAGGGCCGACCAATACAGCACC** |
| **ngsMA21680-2** | **ACAGTGAAGCGGAAAACAGAGCCGAG** | **GCCAATGGGCCGACCAATACAGCACC** |
| **ngsMA21680-3** | **CAGATCAAGCGGAAAACAGAGCCGAG** | **CTTGTAGGGCCGACCAATACAGCACC** |
| **ngsMA21680-4** | **ATCACGAAGCGGAAAACAGAGCCGAG** | **TTAGGCGGGCCGACCAATACAGCACC** |
| **ngsMA21680-5** | **ACTTGAAAGCGGAAAACAGAGCCGAG** | **GATCAGGGGCCGACCAATACAGCACC** |
| **ngsMA21680-6** | **TAGCTTAAGCGGAAAACAGAGCCGAG** | **GGCTACGGGCCGACCAATACAGCACC** |
| **ngsMA21680-7** | **AGTCAAAAGCGGAAAACAGAGCCGAG** | **AGTTCCGGGCCGACCAATACAGCACC** |
| **ngsMA21680-8** | **ATGTCAAAGCGGAAAACAGAGCCGAG** | **CCGTCCGGGCCGACCAATACAGCACC** |
| **ngsMA21680-9** | **GTAGAGAAGCGGAAAACAGAGCCGAG** | **GTCCGCGGGCCGACCAATACAGCACC** |
| **ngsMA21680-wt** | **GTGAAAAAGCGGAAAACAGAGCCGAG** | **GTGGCCGGGCCGACCAATACAGCACC** |
