# Supplementary material for: Establishment of a PEG-mediated protoplast transformation system based on DNA and CRISPR/Cas9 ribonucleoprotein complexes for banana
Source: BMC Plant Biol. 2020 Sep 15;20:425. doi: 10.1186/s12870-020-02609-8 (PMC7493974; doi:10.1186/s12870-020-02609-8)
Supplement: Supplementary file 7 — Additional file 7: Table S7. Primer pairs used to construct OsU3p-PDS. [file 12870_2020_2609_MOESM7_ESM.docx]

**Additional file 7：Table S7. Primer pairs used to construct OsU3p-PDS**

| **OsU3p-PDS** | **gRNA (24 bp)（Primer_F）** | **gRNA (24 bp)（Primer_R）** |
| --- | --- | --- |
| **MAPDSt1** | **ggcgGGCTCCAATTTGGTTGCTTA** | **aaacTAAGCAACCAAATTGGAGCC** |
| **MAPDSt2** | **ggcgTTTTCTGCAAAGACTTCCCG** | **aaacCGGGAAGTCTTTGCAGAAAA** |
| **MAPDSt3** | **ggcgCATCTTTCTGCAATGGTCCA** | **aaacTGGACCATTGCAGAAAGATG** |
| **MAPDSt4** | **ggcgACTTCATCATTGACTCGGTC** | **aaacGACCGAGTCAATGATGAAGT** |
| **MAPDSt5** | **ggcgCATGAGATCCATTGTTCTGC** | **aaacGCAGAACAATGGATCTCATG** |
| **MAPDSt6** | **ggcgCAAGCTTATGTGGAGGCGC** | **aaacGCGCCTCCACATAAGCTTG** |
| **MAPDSt7** | **ggcgATAAGCTTGCCCTCCAAGCA** | **aaacTGCTTGGAGGGCAAGCTTAT** |
| **MAPDSt8** | **ggcgTTGTCCTTAAGCAACCAAAT** | **aaacATTTGGTTGCTTAAGGACAA** |
| **MAPDSt9** | **ggcgAGACATGTCCGTCACATGCA** | **aaacTGCATGTGACGGACATGTCT** |
