# Supplementary material for: Establishment of a PEG-mediated protoplast transformation system based on DNA and CRISPR/Cas9 ribonucleoprotein complexes for banana
Source: BMC Plant Biol. 2020 Sep 15;20:425. doi: 10.1186/s12870-020-02609-8 (PMC7493974; doi:10.1186/s12870-020-02609-8)
Supplement: Supplementary file 8 — Additional file 8: Table S8. Primer pairs used to construct Cas12a-PDS. [file 12870_2020_2609_MOESM8_ESM.docx]

**Additional file 8：Table S8. Primer pairs used to Construct Cas12a-PDS**

| **Cas12a** | **gRNA (27 bp)（Primer_F）** | **gRNA (27 bp)（Primer_R）** |
| --- | --- | --- |
| **MACPFPDSt1** | **agatGATGCCACCAGTATCTTCTCTGA** | **ggccTCAGAGAAGATACTGGTGGCATC** |
| **MACPFPDSt2** | **agatACAAATGTTCTGGAAGCAACAAA** | **ggccTTTGTTGCTTCCAGAACATTTGT** |
| **MACPFPDSt3** | **agatAAAATGAAGGCTCCAATTTGGTT** | **ggccAACCAAATTGGAGCCTTCATTTT** |
| **MACPFPDSt4** | **agatTGCAATGGTCCACGGCCAAGAAA** | **ggccTTTCTTGGCCGTGGACCATTGCA** |
| **MACPFPDSt5** | **agatCACCCAGGACATCTCTAGCCTCC** | **ggccGGAGGCTAGAGATGTCCTGGGTG** |
| **MACPFPDSt6** | **agatGGGAACTTGGTATCAATGATCGC** | **ggccGCGATCATTGATACCAAGTTCCC** |
| **MACPFPDSt7** | **agatGCAGATTCGATTTCCCAGAAACT** | **ggccAGTTTCTGGGAAATCGAATCTGC** |
| **MACPFPDSt8** | **agatCCAGAAACTCTTCCTGCACCTTT** | **ggccAAAGGTGCAGGAAGAGTTTCTGG** |
| **MACPFPDSt9** | **agatCAATATTAAGAAATAGTGAAATG** | **ggccCATTTCACTATTTCTTAATATTG** |
| **MACPFPDSt10** | **agatTCTGGCCAAGTCAGCATTTCACT** | **ggccAGTGAAATGCTGACTTGGCCAGA** |
| **MACPFPDSt11** | **agatCACTTGGACTTTTGCCAGCCATG** | **ggccCATGGCTGGCAAAAGTCCAAGTG** |
