# Supplementary material for: Establishment of a PEG-mediated protoplast transformation system based on DNA and CRISPR/Cas9 ribonucleoprotein complexes for banana
Source: BMC Plant Biol. 2020 Sep 15;20:425. doi: 10.1186/s12870-020-02609-8 (PMC7493974; doi:10.1186/s12870-020-02609-8)
Supplement: Supplementary file 9 — Additional file 9: Table S9. Primer pairs used for in vitro transcription of sgRNA. [file 12870_2020_2609_MOESM9_ESM.docx]

**Additional file 9：Primer pairs used for in vitro transcription of sgRNA.**

| **sgRNA** | **PCR primer(vitro transcription template)** |
| --- | --- |
| **vtr-MAPDSt1F** | **TTAATACGACTCACTATAGGCTCCAATTTGGTTGCTTAgttttag** |
| **vtr-MAPDSt2F** | **TTAATACGACTCACTATAGGTTTTCTGCAAAGACTTCCCGgttttag** |
| **vtr-MAPDSt3F** | **TTAATACGACTCACTATAGGCATCTTTCTGCAATGGTCCAgttttag** |
| **vtr-MAPDSt4F** | **TTAATACGACTCACTATAGGaCTTCATCATTGACTCGGTCgttttag** |
| **vtr-MAPDSt5F** | **TTAATACGACTCACTATAGGCATGAGATCCATTGTTCTGCgttttag** |
| **vtr-MAPDSt6F** | **TTAATACGACTCACTATAGGCAAGCTTATGTGGAGGCGCgttttag** |
| **vtr-MAPDSt7F** | **TTAATACGACTCACTATAGGATAAGCTTGCCCTCCAAGCAgttttag** |
| **vtr-MAPDSt8F** | **TTAATACGACTCACTATAGgTTGTCCTTAAGCAACCAAATgttttag** |
| **vtr-MAPDSt9F** | **TTAATACGACTCACTATAGGAGACATGTCCGTCACATGCAgttttag** |
| **vtr-MAsgRg2R** | **AAGCACCGACTCGGTGCCACT** |
