# Supplementary material for: Establishment of a PEG-mediated protoplast transformation system based on DNA and CRISPR/Cas9 ribonucleoprotein complexes for banana
Source: BMC Plant Biol. 2020 Sep 15;20:425. doi: 10.1186/s12870-020-02609-8 (PMC7493974; doi:10.1186/s12870-020-02609-8)
Supplement: Supplementary file 10 — Additional file 10: Table S10. Primer pairs used for capture-sequencing of Cas9 and RNP system. [file 12870_2020_2609_MOESM10_ESM.docx]

**Additional file 10：Table S10. Primer pairs used for capture-sequencing of Cas9 and RNP system**

| **OsU3p-PDS** | **Primer_F** | **Primer_R** |
| --- | --- | --- |
| **MAPDS1t1-3,8** | **TAAGAATATAGAGGCCAGCATGAACATTATCGG** | **TCACTCCATATACATGTTGGCATCTTTAGCA** |
| **MAPDS1t4** | **ATATAAACATGTCATTTCGTGTCAACCAGT** | **TTTGTGCATTTTGTTCGATATGAATATGGACT** |
| **MAPDS1t5** | **CAGTGGAAACTTCTTTCTGAGTATTTTGAGAGG** | **TTCACATATATTGGTTTGATAGCTCACCGTA** |
| **MAPDS1t6-7** | **CAAGCCAGGAGAGTTTAGCAGATTCGATTTCC** | **CCCGTTCAAACTTATCAACATTAAAGCACAAGACCATAG** |
| **MAPDS1t9** | **AGAACACCTATGACCATCTTCTATTCAGC** | **GTTGAATTGGATCCCACAGGAGCACACT** |
| **ngsMAPDSt1** | **ACTTGAGATACTGGTGGCATCCAAATCCTGA** | **TGACCAAAGCTTGGCAATACAGAAAAACTAAAGGAG** |
| **ngsMAPDSt2** | **ACTTGATGATTTATCACCGGGAAATAGTGGA** | **TGACCACTAAAGATATCAGAATGTCATACCTGCAC** |
| **ngsMAPDSt3** | **ATCACGTGATTTATCACCGGGAAATAGTGGA** | **GATCAGCTAAAGATATCAGAATGTCATACCTGCAC** |
| **ngsMAPDSt4** | **ACTTGACTTTGCTAAGTTAATATGTTCTTTGATAAGTG** | **TGACCAAAAACGGTTCAAAGCAATTAATACACA** |
| **ngsMAPDSt5** | **ACTTGAATCACCAGCTTAATAGGAAATTTTTCTAGAAC** | **TGACCATCTCATCAGGAAATAGCTTAGCCAGT** |
| **ngsMAPDSt6** | **ACTTGATGCAATATTAAGAAATAGTGAAATGCTGA** | **TGACCATCAAATTGAAAAAGATGGCTATATTTCGG** |
| **ngsMAPDSt7** | **ATCACGTGCAATATTAAGAAATAGTGAAATGCTGA** | **GATCAGTCAAATTGAAAAAGATGGCTATATTTCGG** |
| **ngsMAPDSt8** | **ATCACGGATACTGGTGGCATCCAAATCCTGA** | **GATCAGAAGCTTGGCAATACAGAAAAACTAAAGGAG** |
| **ngsMAPDSt9** | **ACTTGAGTGTTCCACATTGTTTCCTTCCAG** | **TGACCACAAAATTAAGAGTCAACAAACTAAGGTG** |
| **ngsMAPDSwt1** | **ACAGTGGATACTGGTGGCATCCAAATCCTGA** | **CAGATCAAGCTTGGCAATACAGAAAAACTAAAGGAG** |
| **ngsMAPDSwt2** | **ACAGTGTGATTTATCACCGGGAAATAGTGGA** | **CAGATCCTAAAGATATCAGAATGTCATACCTGCAC** |
| **ngsMAPDSwt3** | **ATGTCATGATTTATCACCGGGAAATAGTGGA** | **GTAGAGCTAAAGATATCAGAATGTCATACCTGCAC** |
| **ngsMAPDSwt4** | **ACAGTGCTTTGCTAAGTTAATATGTTCTTTGATAAGTG** | **CAGATCAAAACGGTTCAAAGCAATTAATACACA** |
| **ngsMAPDSwt5** | **ACAGTGATCACCAGCTTAATAGGAAATTTTTCTAGAAC** | **CAGATCTCTCATCAGGAAATAGCTTAGCCAGT** |
| **ngsMAPDSwt6** | **ACAGTGTGCAATATTAAGAAATAGTGAAATGCTGA** | **CAGATCTCAAATTGAAAAAGATGGCTATATTTCGG** |
| **ngsMAPDSwt7** | **ATGTCATGCAATATTAAGAAATAGTGAAATGCTGA** | **GTAGAGTCAAATTGAAAAAGATGGCTATATTTCGG** |
| **ngsMAPDSwt8** | **ATGTCAGATACTGGTGGCATCCAAATCCTGA** | **GTAGAGAAGCTTGGCAATACAGAAAAACTAAAGGAG** |
| **ngsMAPDSwt9** | **ACAGTGGTGTTCCACATTGTTTCCTTCCAG** | **CAGATCCAAAATTAAGAGTCAACAAACTAAGGTG** |
