# Supplementary material for: Establishment of a PEG-mediated protoplast transformation system based on DNA and CRISPR/Cas9 ribonucleoprotein complexes for banana
Source: BMC Plant Biol. 2020 Sep 15;20:425. doi: 10.1186/s12870-020-02609-8 (PMC7493974; doi:10.1186/s12870-020-02609-8)
Supplement: Supplementary file 11 — Additional file 11: Table S11. Primer pairs used for capture-sequencing of Cas12a system. [file 12870_2020_2609_MOESM11_ESM.docx]

**Additional file 11：Table S11. Primer pairs used for capture-sequencing of Cas12a system**

| **Cas12a** | **Primer_F** | **Primer_R** |
| --- | --- | --- |
| **MACPF1PDS1-5** | **TGTTTCTTCTTTACATTCTACCCGTTGTTCC** | **GGCACATGTTTATGGATCATAGTACAAGTGG** |
| **MACPFPDS6-11** | **GAATGCCCATATATCTGGACAAATGCTT** | **GCAGTCATTTGAACTTTGATCTAAGCA** |
| **ngsMACPFPDSt1** | **CGATGTTCGATTGTAGATAGACCTGC** | **CGATGTCAGTTTGTTGCTTCCAGAAC** |
| **ngsMACPFPDSt2** | **CAGATCCATTATCGGATCTGTCTCTC** | **CTTGTATTTGAAACCCATGAACTCAC** |
| **ngsMACPFPDSt3** | **ACTTGAGTGGAACAATTCAGAGAAGA** | **GATCAGGAGAGGGCTGGCACCATGTC** |
| **ngsMACPFPDSt4** | **AGTCAATTCTGCAAAGACTTCCCGAG** | **AGTTCCGATATTCTCTGCATTTAGGT** |
| **ngsMACPFPDSt5** | **GTAGAGCTTCTGGTAGGTCTGGCTGG** | **GTAGAGCATATCATAGATTCAAAGCC** |
| **ngsMACPFPDSt6** | **GTTTCGGAAGTTGGGGCATATCCCAA** | **CGTACGTTCTGGGAAATCGAATCTGC** |
| **ngsMACPFPDSt7** | **ACTGATGGTATCAATGATCGCTTGCA** | **ATGAGCTCACATTCTAATTACACGAA** |
| **ngsMACPFPDSt8** | **CAACTAGGTATCAATGATCGCTTGCA** | **CACCGGTCACATTCTAATTACACGAA** |
| **ngsMACPFPDSt9** | **CAGGCGACTTCTGCAAATTAGTGGTG** | **CATGGCGCTTGCCCTCCAAGCATGGC** |
| **ngsMACPFPDSt10** | **CGGAATACTTCTGCAAATTAGTGGTG** | **CTAGCTGCTTGCCCTCCAAGCATGGC** |
| **ngsMACPFPDSt11** | **GCGCTAGAAATAGTGAAATGCTGACT** | **TAATCGAAGATGGCTATATTTCGGTA** |
| **ngsMACPFPDS1wt** | **ACAGTGTCGATTGTAGATAGACCTGC** | **GCCAATCAGTTTGTTGCTTCCAGAAC** |
| **ngsMACPFPDS2wt** | **ATCACGCATTATCGGATCTGTCTCTC** | **TTAGGCTTTGAAACCCATGAACTCAC** |
| **ngsMACPFPDS3wt** | **TAGCTTGTGGAACAATTCAGAGAAGA** | **GGCTACGAGAGGGCTGGCACCATGTC** |
| **ngsMACPFPDS4wt** | **ATGTCATTCTGCAAAGACTTCCCGAG** | **CCGTCCGATATTCTCTGCATTTAGGT** |
| **ngsMACPFPDS5wt** | **GTGAAACTTCTGGTAGGTCTGGCTGG** | **GTGGCCCATATCATAGATTCAAAGCC** |
| **ngsMACPFPDS6wt** | **GAGTGGGAAGTTGGGGCATATCCCAA** | **GGTAGCTTCTGGGAAATCGAATCTGC** |
| **ngsMACPFPDS7wt** | **ATTCCTGGTATCAATGATCGCTTGCA** | **CAAAAGTCACATTCTAATTACACGAA** |
| **ngsMACPFPDS8wt** | **CACGATGGTATCAATGATCGCTTGCA** | **CACTCATCACATTCTAATTACACGAA** |
| **ngsMACPFPDS9wt** | **CATTTTACTTCTGCAAATTAGTGGTG** | **CCAACAGCTTGCCCTCCAAGCATGGC** |
| **ngsMACPFPDS10wt** | **CTATACACTTCTGCAAATTAGTGGTG** | **CTCAGAGCTTGCCCTCCAAGCATGGC** |
| **ngsMACPFPDS11wt** | **TACAGCGAAATAGTGAAATGCTGACT** | **TATAATAAGATGGCTATATTTCGGTA** |
